# Decreased vascular smooth muscle contractility in Hutchinson–Gilford Progeria Syndrome linked to defective smooth muscle myosin heavy chain expression

**DOI:** 10.1038/s41598-021-90119-4

**Published:** 2021-05-19

**Authors:** Ryan von Kleeck, Paola Castagnino, Emilia Roberts, Shefali Talwar, Giovanni Ferrari, Richard K. Assoian

**Affiliations:** 1 Department of Systems Pharmacology and Translational Therapeutics, University of Pennsylvania, Philadelphia, PA 19104 USA; 2 Center for Engineering MechanoBiology, University of Pennsylvania, Philadelphia, PA 19104 USA; 3 Institute of Translational Medicine and Therapeutics at University of Pennsylvania, Philadelphia, PA 19104 USA; 4 Departments of Surgery and Biomedical Engineering, Columbia University, New York, NY 10032 USA

**Keywords:** Biochemistry, Cardiovascular biology, Myosin

## Abstract

Children with Hutchinson–Gilford Progeria Syndrome (HGPS) suffer from multiple cardiovascular pathologies due to the expression of progerin, a mutant form of the nuclear envelope protein Lamin A. Progerin expression has a dramatic effect on arterial smooth muscle cells (SMCs) and results in decreased viability and increased arterial stiffness. However, very little is known about how progerin affects SMC contractility. Here, we studied the LaminA^G609G/G609G^ mouse model of HGPS and found reduced arterial contractility at an early age that correlates with a decrease in smooth muscle myosin heavy chain (SM-MHC) mRNA and protein expression. Traction force microscopy on isolated SMCs from these mice revealed reduced force generation compared to wild-type controls; this effect was phenocopied by depletion of SM-MHC in WT SMCs and overcome by ectopic expression of SM-MHC in HGPS SMCs. Arterial SM-MHC levels are also reduced with age in wild-type mice and humans, suggesting a common defect in arterial contractility in HGPS and normal aging.

## Introduction

Hutchinson–Gilford Progeria Syndrome (HGPS) is a rare premature aging disease. The “classical” form of HGPS is caused by a point mutation (c. 1824C>T; pG608G) in the *LMNA* gene (Lamin A)^1^. This mutation causes the expression of progerin, a mutant form of the nuclear envelope protein Lamin A that has a 50 amino acid deletion and retains a farnesylated C-terminus^1,2^. Others have shown that progerin can alter nuclear lamina structure to cause nuclear blebbing, altered nuclear import and export, and dynamic epigenetic changes^1,3–5^. Children with HGPS begin to display clinical symptoms about two years after birth, including loss of hair, thin and aged skin, and growth retardation—characteristics of normally aged individuals^1,6^. Cardiovascular complications, including arterial fibrosis, generation of severe atherosclerotic lesions, and the death of arterial smooth muscle cells (SMCs) are hallmarks of the disease, and HGPS children typically die from heart attacks or strokes^7–11^. Vascular SMCs normally populate the medial layer of arteries, but at autopsy, HGPS patients display very few medial SMCs, and their arteries are instead filled with a collagen-dense matrix^9,11^.

One of the main functions of vascular SMCs is to regulate blood flow to target organs by modulating arterial tone and peripheral resistance. Vascular tone is regulated by periodic contraction and dilation of the arteries^12^. Similar to other muscle cells, the major contractile components in SMCs consists of actin and myosin^13,14^. However, a smooth muscle-specific myosin heavy chain (SM-MHC, encoded by the *Myh11* gene) and smooth muscle alpha-actin (SMA, encoded by the *Acta2* gene) exist in SMCs, and these play important roles in SMC contractility^13–16^.

The *Myh11* gene is alternatively spliced into four SM-MHC isoforms, and these allow for the prolonged tonic contraction of SMCs^17^. Additionally, SMCs contain other cell-specific contractile proteins such as transgelin (gene *Tagln*), an actin cross-linking protein, and calponin (gene *Cnn1*) that can regulate ATPase activity of myosin in smooth muscle^18–20^. The inability to properly regulate vascular tone through contraction and dilation may lead to impaired blood flow to target tissues^12,21^.

As HGPS is a rare disease, animal models are used to understand the pathology caused by progerin expression, especially when looking for early events that are not amenable to analysis at autopsy. The Lmna^G609G/G609G^ mouse model (hereafter referred to as HGPS mice) has been commonly used since it contains the equivalent point mutation seen in the classical HGPS human syndrome at the mouse *Lmna* locus^22^. This mouse model recapitulates many of the cardiovascular complications seen in the human syndrome including arterial fibrosis and stiffening as well as the progressive loss of aortic SMCs as these mice approach the end of their lifespan^22–24^. Studies in HGPS mice have also observed decreased arterial vasoconstriction, although these studies were performed on arteries isolated from older HGPS mice (approx.100 and 140 days) that had the expected loss of SMCs at 140 days^23,25^. Thus, whether reduced arterial contractility is an early event in HGPS remains unclear.

In this work, we analyzed arterial gene expression and contractility in young (2-month) HPGS mice, before SMC loss is detectable^26^, with the goal of identifying potentially causal events in HPGS pathogenesis. We found a reduced abundance of SM-MHC mRNA and protein that was associated with impaired arterial contraction in isolated HGPS carotid arteries and reduced force generation in isolated HGPS SMCs. Moreover, RNAi-mediated downregulation of SM-MHC reduced traction forces in WT SMCs while enforced expression of SM-MHC increased traction force in HGPS SMCs. Interestingly, reduced SM-MHC abundance is also a characteristic of normal arterial aging, both in WT mice and in humans. Impaired arterial SMC contractility may be a common feature of premature and normal aging.

## Results

### Reduced smooth muscle myosin heavy chain and contractility early in HGPS

**Figure 1:**
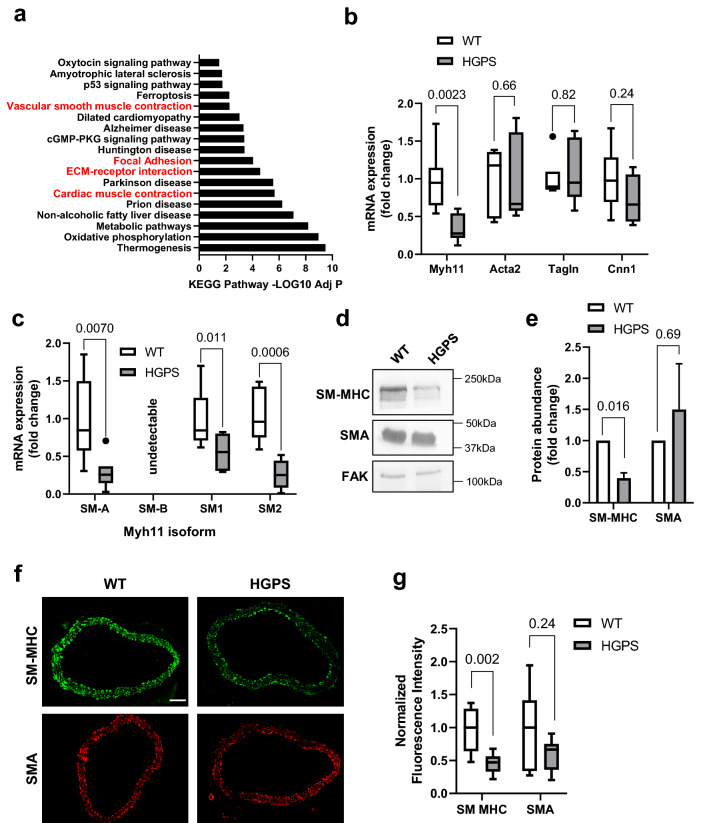
Reduced smooth muscle myosin heavy chain abundance in HGPS aortas. (**a**) KEGG pathway analysis was performed on the differentially expressed genes from 2-month WT versus HGPS descending aortas (n = 6 per genotype). KEGG pathways with p-adjusted values < 0.05 are shown. (**b**) Descending aortas were isolated from 2-month WT and HGPS mice (n = 5–7 per genotype). SMC contractility gene transcript levels were assessed by RT-qPCR and plotted relative to the WT control. (**c**) Smooth muscle myosin heavy chain isoform transcript expression was assessed by RT-qPCR in 2-month WT and HGPS descending aortas (n = 7 per genotype) and plotted relative to the WT control. (**d**) Representative images of SM-MHC and SMA protein levels determined from 2-month WT and HGPS descending aortas by immunoblotting. Focal Adhesion Kinase (FAK) is shown as the loading control. Black boxes around the immunoblots indicate cropping for removal of extraneous space. Unprocessed blots are shown in Fig. S1. (**e**) Quantification of immunoblotting results normalized for loading and plotted relative to the WT control. Results show mean ± SE (n = 7 per genotype). (**f**) Representative images of 2-month WT (n = 7) and HGPS (n = 9) descending aorta cross sections immunostained for SM-MHC and SMA; scale bar = 50 µm. (**g**) Quantification of aortic immunostaining with results plotted relative to the WT controls. Results in b, c and g are shown as box plots with Tukey whiskers, and significance was determined by Mann–Whitney tests. Significance in panel e was determined by Wilcoxon signed rank test and data is displayed as mean ± SE.

To better understand the effects of progerin expression on SMC function, we interrogated our recent genome-wide transcriptome analysis of isolated descending aortas from 2-month-old WT and HGPS mice^26^. We chose to focus on mice at 2-months of age as, aside from stunted growth, these young mice do not show overt features of the syndrome. In particular, the arteries of 2-month HGPS mice have yet to show SMC loss or apoptosis^26^. KEGG pathway analysis^27^ of the differentially expressed genes in these HGPS vs. WT aortas indicated that several pathways associated with contractility were altered early in HGPS including vascular smooth muscle contraction, focal adhesions, and ECM-receptor interactions (Fig. 1a; red).

To extend the results of the KEGG analysis, we performed RT-qPCR on 2-month WT and HGPS aortas and evaluated the expression of several SMC contractile genes (Fig. 1b). The mRNA level of *Myh11*, the gene encoding SM-MHC, was significantly decreased while levels of other contractility-regulating genes (*Acta2, Tagln, and Cnn1)* mRNA were not significantly altered (Fig. 1b). SM-MHC has multiple isoforms^15,17^, and we found that all the *Myh11* mRNA isoforms present in the aorta were decreased in HGPS (Fig. 1c). Additionally, immunoblotting (Fig. 1d–e) and immunostaining (Fig. 1f–g) of 2-month WT and HGPS aortas showed decreased abundance of SM-MHC protein while SMA protein was minimally affected.

**Figure 2:**
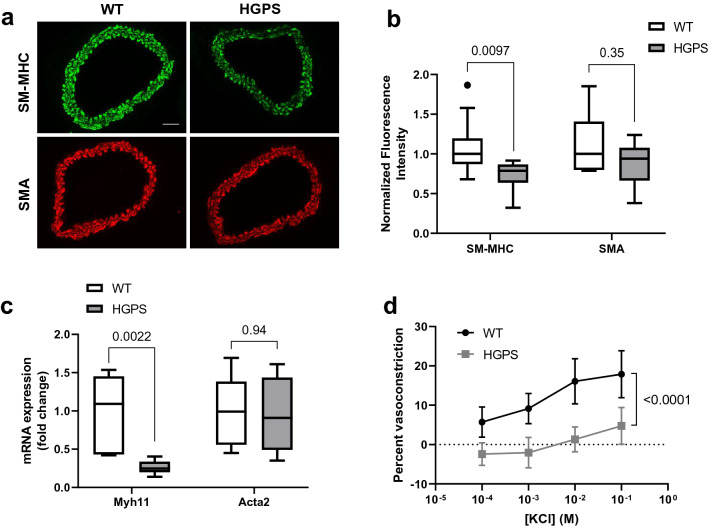
Reduced smooth muscle myosin heavy chain and decreased contractility in HGPS carotid arteries. (**a**) Representative images of 2-month WT (n = 10) and HGPS (n = 7) carotid artery cross sections immunostained for SM-MHC and SMA; scale bar = 50 µm. (**b**) Quantification of carotid artery immunostaining with results plotted relative to the WT controls. (**c**) Carotid arteries from 2-month WT and HGPS mice (n = 6 per genotype) were isolated, and *Myh11* and *Acta2* mRNA transcript levels were determined by RT-qPCR. Results are plotted relative to the WT controls. (**d**) Mixed sex 2-month WT (n = 7) and HGPS (n = 5) carotid arteries were mounted on a pressure myograph and pressurized to 90 mm Hg. Percent vasoconstriction was measured in response to increasing concentrations of KCl. Results in b and c are plotted as box plots with Tukey whiskers, and significance was determined by Mann–Whitney tests. Significance in panel d was determined by two-way ANOVA, and results show mean ± SD.

We then expanded our analysis to the carotid artery and found similar results to what we observed in the aorta: HGPS carotid arteries showed a preferential decrease in the level of SM-MHC protein as determined by immunostaining (Fig. 2a,b) and a decrease in *Myh11* mRNA as determined by RT-qPCR (Fig. 2c). The reduced expression of SM-MHC led us to reason that arterial contractility might be defective in HGPS arteries, even at early ages when the number of arterial SMCs is still similar to WT^26^. To test this hypothesis, we measured how 2-month WT and HGPS carotid arteries respond to KCl, a canonical vasoconstrictor dependent on SM-MHC for its effect^14^. Contractility was measured on a pressure myograph so that results could be obtained at physiological arterial pressure and stretch^28,29^. Indeed, carotid artery vasoconstriction to KCl was reduced in HGPS (Fig. 2d), supporting the idea that decreased vasoconstriction is an early event in HGPS and even precedes the loss of SMCs from large arteries.

### Reduced SM-MHC is causal for traction force defects in HGPS SMCs

**Figure 3:**
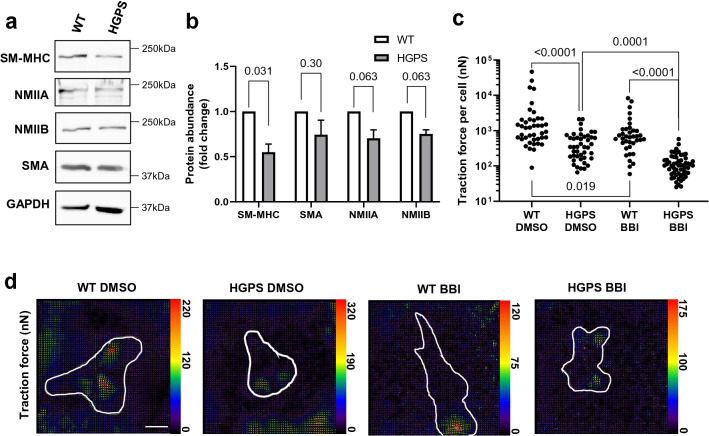
Decreased myosin abundance and traction force in isolated HGPS aortic vascular smooth muscle cells. (**a**) Representative images of cytosolic lysates from cultured SMCs immunoblotted for SM-MHC, non-muscle myosin II A and B (NMIIA and NMIIB, respectively), and SMA. GAPDH is shown as the loading control. Black boxes around the immunoblots indicate cropping for removal of extraneous space. Unprocessed blots are shown in Fig. S1. Blots were stripped and re-probed when evaluating the abundance of multiple myosins and SMA. (**b**) Quantification of immunoblotting results normalized for loading and plotted relative to the WT control. Results show mean ± SE (n = 5–7). Significance was determined by Wilcoxon signed rank test. (**c**) Cultured SMCs were incubated on polyacrylamide hydrogels for 24 h. Cells were treated with vehicle (DMSO) or 15 µM Blebbistatin (BBI) for 30 min prior to traction force analysis. Total traction force plots (n = 37–57 cells) are shown in panel c, and each dot represents the total traction force of a single cell. Significance was determined by Mann–Whitney tests, and results are displayed as scatter plots. (**d**) Representative traction force images of cells analyzed in panel c. Cells areas are outlined in white, and traction force scale bars (in nanonewtons) are displayed to the right of each respective image. Optical scale bar = 50 µm.

To determine if the reduced arterial contractility observed in HGPS carotids was associated with defective vascular SMC force generation, we isolated primary aortic SMCs from 2-month WT and HGPS mice. First, we evaluated SM-MHC and SMA abundance in isolated SMCs and observed a preferential reduction in SM-MHC levels in HGPS, consistent with our observations in vivo (Fig. 3a,b). Although smooth muscle myosins are thought to be the major force generating myosin in SMCs, non-muscle myosins IIA and IIB (NMII2A and NMIIB) are also present and are believed to perform a house-keeping function^30^. The abundance of these non-muscle myosins trended lower in HGPS SMCs although not to the same extent as SM-MHC (Fig. 3a,b).

We next performed traction force microscopy on primary WT and HGPS SMCs. HGPS SMCs presented with decreased force generation compared to WT (Fig. 3c,d; column 1 vs 2), suggesting an important role for SM-MHC in contractility. We then assessed the contribution of non-muscle myosins to HGPS cell force generation by treating WT and HGPS SMCs with blebbistatin, a myosin-ATPase inhibitor (Fig. 3c,d). We used a low concentration of blebbistatin expected to inhibit non-muscle myosins but not SM-MHC^31^. This low dose treatment decreased traction force in HGPS SMCs to very low levels (Fig. 3c; column 2 vs 4). Low dose blebbistatin treatment also reduced traction force in WT SMCs (Fig. 3c; columns 1 vs 3), but the remaining traction force in WT SMCs remained much higher than in the blebbistatin-treated HGPS cells, (Fig. 3c; columns 3 vs 4) as would be expected given the higher level of SM-MHC in WT SMCs. Note that the magnitude of the blebbistatin effect on decreasing mean traction force was similar in WT and HGPS SMCs (~ 70 and 75%, respectively) despite their difference in SM-MHC abundance. This finding supports the notion that our low dose blebbistatin treatment was preferentially targeting the non-muscle myosins and not smooth muscle myosin as reported^31^ (also see “Discussion”). The data in Fig. 3 therefore indicate that defective expression of SM-MHC in HGPS SMCs correlates with reduced cell traction force and that HGPS SMCs maintain a basal level of force generation through the action of non-muscle myosins.

**Figure 4:**
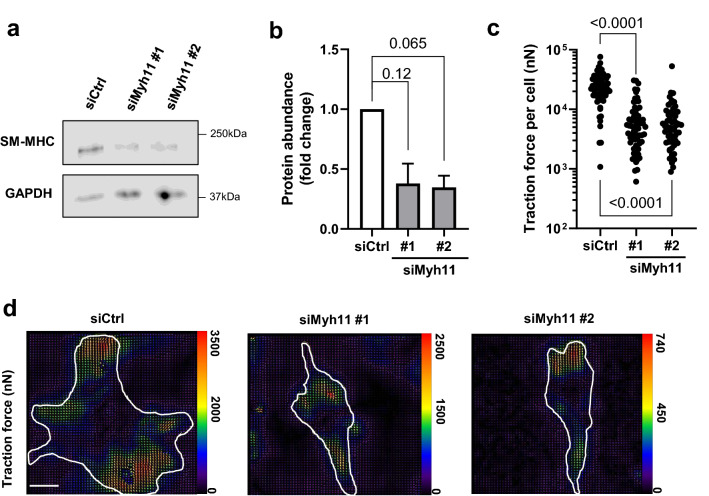
Knock down of SM-MHC reduces force generation in WT SMCs. Aortic SMCS isolated from 2-month WT mice were incubated with a control siRNA (siCtrl) or two distinct siRNAs targeting Myh11. (**a**) Representative images of lysates from the transfected cells immunoblotted for SM-MHC and GAPDH (loading control). Black boxes around the immunoblots indicate cropping for the removal of extraneous space. Unprocessed blots are shown in Fig. S1. (**b**) Quantification of immunoblotting results normalized for loading and plotted relative to the siCtrl control**.** Results show mean ± SE (n = 4–5). Significance was determined by Wilcoxon signed rank test. (**c**) Total traction force plots of siCtrl- or siMyh11-treated SMCs (n = 63–69 cells). Each dot represents the total traction force of a single cell. Significance was determined by Mann–Whitney tests. (**d**) Representative traction force images of cells analyzed in panel c. Cell areas are outlined in white, and traction force scale bars (in nanonewtons) is displayed to the right of each respective image. Optical scale bar = 50 µm.

**Figure 5:**
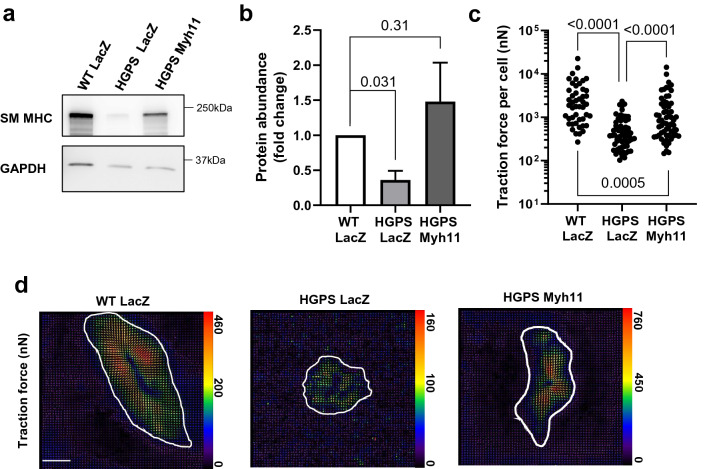
Smooth muscle myosin heavy chain overexpression restores contractility in isolated HGPS smooth muscle cells. Aortic SMCs isolated from 2-month WT and HGPS mice were incubated with adenovirus encoding LacZ (control) or *Myh11* for 72 h. (**a**) Representative images of lysates from the infected cells immunoblotted for SM-MHC and GAPDH (loading control). Black boxes around the immunoblots indicate cropping for the removal of extraneous space. Unprocessed blots are shown in Fig. S1. (**b**) Quantification of immunoblotting results normalized for loading and plotted relative to the WT LacZ control**.** Results show mean ± SE (n = 6). Significance was determined by Wilcoxon signed rank test. (**c**) Total traction force plots of primary WT and HGPS aortic SMCs incubated with an adenovirus encoding LacZ or *Myh11* (n = 47–65 cells). Each dot represents the total traction force of a single cell. Significance was determined by Mann–Whitney tests. (**d**) Representative traction force images of cells analyzed in panel c. Cell areas are outlined in white, and traction force scale bars (in nanonewtons) are displayed to the right of each respective image. Optical scale bar = 50 µm.

We then treated WT SMCs with two distinct siRNAs targeting *Myh11* (Fig. 4a,b) and found that each siRNA resulted in ~ 70% reduction in mean traction force (Fig. 4c,d), which was similar to the ~ 80% reduction we observed in HGPS SMCs as compared to WT (Fig. 3c; columns 1 vs. 2). Thus, deliberate reduction of SM-MHC in WT SMCs phenocopied the reduced traction force seen in HGPS SMCs. Conversely, adenoviral enforced expression of SM-MHC (Fig. 5a,b) increased traction force in HGPS SMCs (Fig. 5c,d). We do note that traction forces in the HPGS SMCs infected with adeno-*Myh11* were not quite increased to the levels seen in WT SMCs (see “Discussion”). Nevertheless, the results collectively indicate that defective SM-MHC expression in HGPS SMCs is causally related to the defect in SMC force generation.

### Reduced SM-MHC in aging aortas of WT mice and humans

**Figure 6:**
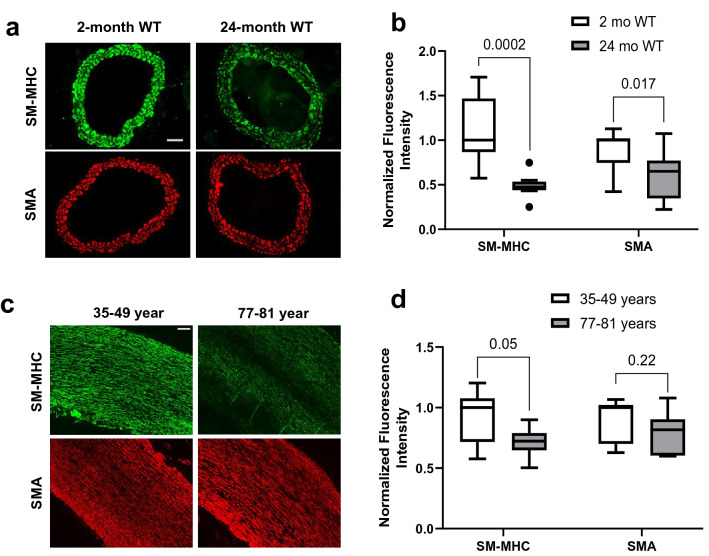
Decreased smooth muscle myosin heavy chain in aged mouse and human arteries. (**a**) Representative images of 2-month (n = 7) and 24-month (n = 9–10) WT mice carotid artery cross sections immunostained for SM-MHC and SMA; scale bar = 50 µm. (**b**) Quantification of carotid artery immunostaining with results plotted relative to the WT controls. (**c**) Representative images of human ascending aorta and aortic root cross sections of young (35–49 years, n = 6–7) and old (77–81 years, n = 7) immunostained for SM-MHC and SMA; scale bar = 200 µm. (**d**) Quantification of immunostaining of ascending aorta and aortic root sections with results plotted relative to the young cohort. Results in b and d show box plots with Tukey whiskers. Significance was determined by 1-tail Mann–Whitney tests.

Many parallels have been drawn between HGPS and natural aging, especially in regard to age-associated cardiovascular pathology^1,8,10^. As reduced arterial contractility has also been observed with age^32,33^, we asked if this reduction in contractility was associated with a preferential decrease in SM-MHC. Indeed, immunofluorescence staining of carotid artery cross sections from 2- and 24-month WT mice revealed a decrease in SM-MHC staining in the aged WT mice and a smaller difference in the levels of SMA (Fig. 6a,b). Moreover, we found a similar reduction in SM-MHC levels in ascending aorta and aortic root sections from relatively young (age = 35–49 years) versus older (age = 77–81 years) humans whereas SMA abundance was minimally changed (Fig. 6c,d). Thus, reduced SM-MHC expression, and a consequent effect on arterial contraction, may be a common event in both HGPS and the natural aging process.

## Discussion

Medial SMCs play a major role in arterial vasoconstriction, which is essential for regulation of vascular tone and blood flow to target tissues^12,21^. As SMC death is a hallmark of HGPS, it is not surprising that reduced arterial contractility has been observed in late HGPS, when arteries are deficient in their number of SMCs^9,22,23^. However, we show here that reduced contractility in HGPS begins before the onset of many clinical phenotypes of the syndrome and prior to the loss of SMCs. Thus, reduced vasoconstriction is one of the earliest cardiovascular defects in HGPS, at least in mice, and not merely a secondary consequence of SMC death. We also show that these young HGPS arteries are defective for expression of SM-MHC and that arteries of aged WT mice and humans also display reduced SM-MHC abundance. Thus, the defective expression of SM-MHC in the arteries of young HGPS mice foreshadows the pathophysiology of normal arterial aging.

By using primary SMCs in culture, we were able to link the decreased abundance of SM-MHC to defective force generation. Our initial experiments used pharmacologic inhibition of myosin II with blebbistatin and were based on Limouze et al.^31^, which demonstrated that the blebbistatin IC50 for smooth muscle myosins is at least tenfold greater than that of the non-muscle myosins (80 versus 2–5 µM). Thus, we reasoned that use of 15 µM blebbistatin should be selective for the non-muscle myosins, which are expressed at similar levels in both WT and HGPS SMCs. Though the IC50 difference was smaller, a similar result has also been reported Zhang et al.^34^ We acknowledge that both of these studies examined blebbistatin effects on purified proteins and that the blebbistatin IC50's for different myosins may differ in cells. Nevertheless, if 15 µM blebbistatin were also inhibiting smooth muscle myosin, one would not expect to see the similar effect of blebbistatin on mean traction force that we observed in WT versus HGPS SMCs (given their large difference in the amount of SM-MHC).

Further analysis used RNAi and ectopic expression to assess the effect of SM-MHC on traction force. Reducing SM-MHC levels in WT SMCs with RNAi produced a similar percent reduction in traction force as observed in HGPS SMCs. Additionally, restoration of SM-MHC levels in HGPS through adenoviral overexpression partially rescued SMC force generation. This incomplete effect could be due to a multifaceted regulation of SMC contractility that relies on regulatory proteins and signaling pathways aside from smooth muscle myosin and actin^13,14^. Indeed, KEGG pathway^27^ evaluation of our genome-wide transcriptome analysis (GEO ascension number GSE165409) revealed slight decreases in genes such as *Myl9*, the smooth muscle myosin regulatory light chain, and *Mlck1*, smooth muscle myosin light chain kinase. Although not reduced to the extent of SM-MHC (*Myh11*), the combined contributions of other gene products such as these may be required to restore WT levels of contractility to HGPS SMCs.

Even though we have reported decreased force generation in isolated SMCs, arterial contractility at a tissue level can depend on multiple factors from the external environment^13,14^. For example, the composition and stiffness of the extracellular matrix can dictate the ability of smooth muscle to contract^35,36^, and the arteries of HGPS mice are much stiffer than WT^23,24,26^. However, work done by del Campo et al.^25^, albeit in older (14 weeks) HGPS mice, found that collagen disruption by collagenase in aorta did not restore the contractile response of arteries in response to KCl. Their finding suggests that although the ECM is stiffer in HGPS arteries, it may not play as much of a role in regulating vasoconstriction as altered SMC force generation. Additionally, arterial contractility in vivo relies on complex signaling of agonist-receptor interactions^13,14^. It is possible that in addition to reduced contractile machinery, cells in HGPS arteries are producing altered amounts of circulating factors essential for regulating vasoconstriction and vasodilation.

How progerin affects SM-MHC mRNA levels remains to be clearly defined. Research suggests that incorporation of progerin into the nuclear lamina can change the nuclear architecture, making nuclei stiffer and blebbed^37,38^. This effect could potentially interfere with the nuclear translocation of transcription factors essential for contractile gene transcription. Additionally, cell contractility is tightly tied to mechanotransduction, or the sensing of the mechanical properties of the microenvironment^39^. As transmission of mechanical stimuli from the microenvironment to the nucleus depends on the nuclear lamina and LINC (linker of nucleoskeleton and cytoskeleton) complex^39^, the altered nuclear lamina in HGPS may impair mechanotransduction^37,40^.

Many phenotypic similarities exist between HGPS and normal aging, and it has been reported that some aged tissues show low levels of progerin expression^8,41–43^. We show that SM-MHC levels are also decreased in aging mouse and human arteries. It remains to be determined if the molecular mechanisms underlying reduced SM-MHC abundance in HGPS and normal arterial aging are common or distinct. Nevertheless, if reduced levels of SM-MHC leads to diminished arterial contraction and blood flow, then this phenotype of defective SM-MHC mRNA and protein abundance in early HGPS may provide important insight into complications arising from reduced blood flow in normal aging^44,45^.

## Methods

All methods were carried out in accordance with relevant guidelines and regulations.

### Mice and artery isolations

For aging experiments, WT C57BL/6 mice were purchased from Jackson Labs and aged up to 24-months. LMNA^G609G/+^ mice on the C57/BL6 background were generously provided Dr. Carlos Lopez-Otin (Universidad de Oviedo, Oviedo, Spain)^22^. Mice were genotyped by ear clipping and DNA was isolated using Puregene Core Kit A (Qiagen 1042601) according to manufacturer’s instructions. DNA was amplified using the following primers (at a final concentration of 0.5 μm): Forward: AAGGGGCTGGGAGGACAGAG; and Reverse: AGCATGCCATAGGGTGGAAGGA using the following PCR protocol: 95 °C for 60 s, [95 °C for 30 s, 64 °C for 30 s, 72 °C for 30 s] x 35, 72 °C for 120 s to generate bands of 100bp for WT and 240bp for LMNA^G609G^. Mice were fed a chow diet ad libitum. For experiments including HGPS arteries or cells, WT littermate controls were obtained from LMNA^G609G/+^ matings.

Unless noted otherwise, arteries were perfused with PBS in situ through the left ventricle, the left and right carotids were isolated, and the descending aorta was isolated from the end of the aortic arch to the diaphragm. The isolated arteries were cleaned of excess fat and stored in RNAlater for RT-qPCR, flash frozen and stored at − 80 °C for protein analysis, or fixed in Prefer (Anatech #414) for embedding and sectioning. Animal protocols were approved by the University of Pennsylvania Institutional Animal Care and Use Committee, and animal experiments followed the recommended ARRIVE guidelines. Experiments were performed on male mice unless otherwise specified in the legends.

### Human artery sections

Human aortic root or ascending aorta sections from both men and women were obtained from patients undergoing aortic valve/root replacement and/or ascending aortic repair/replacement at Columbia University Irving Medical Center. Approval was obtained under IRB Protocol # AAAR6796 with appropriate informed consent from all subjects following all ethical guidelines. Specimens were fixed in 10% formalin for 24 h and embedded in paraffin. Cross sections (5 µm) were prepared. Two cohorts of samples (young and old) were generated based on age (35–49 years and 77–81 years, respectively). Results from aortic roots and ascending aortas were combined for data analysis.

### Genome-wide transcript analysis

Our previously described genome-wide analysis of differentially expressed genes in 2-month WT versus HGPS mice^26^ (GEO ascension number GSE165409) was subjected to KEGG pathway analysis^27^ using gprofiler (https://biit.cs.ut.ee/gprofiler/gost). The cutoffs used were > 0.5 log_2_ or < − 0.5 log_2_ fold change and an adjusted p-value of < 0.0001.

### Carotid artery contractility analysis

Carotid artery contractility was measured using a DMT 114P pressure myograph. Dissected left carotid arteries were cleaned of excess fat, secured at both ends to 380-µm diameter steel cannulas in the myograph chamber using four silk sutures (Teleflex Medical, 104-S), and maintained in 5 ml of Hanks Balanced Salt Solution (HBSS) with calcium. The closed system was checked for leaks by pressurizing the vessel at 30 mm Hg using HBSS. The system was then returned to 0 mm Hg, and un-stretched vessel length (UVL) was determined as the minimum distance between the cannulas where the artery was no longer bent.

Mounted vessels were preconditioned by stretching them axially to 1.15 times their UVL at 40 mm Hg for 15 min and then 1.3 times their UVL at 60 mm Hg for 15 min. The preconditioned vessels were brought to their in-vivo stretch (IVS) based on the values we recently reported^26^ and pressurized to 90 mm Hg. Increasing concentrations of KCl (10^–4^ M to 10^–1^ M in HBSS with calcium; 5 ml) were added to the vessel chamber sequentially at 10-min intervals or once the constriction plateau had been reached. Each new KCl solution was added after removing the prior KCl solution. Outer diameters were recorded in real time at each drug concentration using an inline tracking camera (Imaging Source) and MYOVIEW software.

### Cell culture, viral infection, and RNAi

Primary mouse SMCs were isolated from the descending aortas of 2-month WT and HGPS male mice and prepared by explant culture as described^46^. SMCs were cultured in growth medium [1:1 Dulbecco’s modified Eagle’s medium (DMEM)/Ham’s F-12 supplemented with 2 mM l-glutamine and 20 mM HEPES, pH 7.4] with 20% FBS. Cells were passaged at near confluence with trypsin/EDTA and used between passages 4–6. Near confluent, asynchronous SMCs were infected with adenoviruses encoding LacZ (control) or *Myh11* (Vigene Biosciences VH802236). Adenoviruses were amplified using standard procedures, added to WT or HGPS SMCs in growth medium at a MOI of 800, and incubated overnight. The infected cells were washed and incubated for 72 h in fresh growth medium before subsequent analysis.

siRNA-mediated knock down of SM-MHC in near-confluent WT SMCs was performed using Lipofectamine RNAiMAX Transfection Reagent (Thermofisher #13778100) in OPTI-MEM with two distinct siRNAs (Ambion s70252 and s70253) at a final siRNA concentration of 150 nM. A non-specific siRNA (Ambion 4390843) was used as control. After 4 h of siRNA transfection, cells were switched to fresh growth medium. Protein or traction force analysis was performed 72 h after transfection.

### Traction force microscopy (TFM)

Polyacrylamide hydrogels with a Young’s modulus of 20–25 kPa were prepared as described^47^ with 0.2 µm diameter fluorescent microspheres (F8810; Invitrogen, 1% vol/vol) added to the polyacrylamide solution before polymerization. Hydrogels were coated with 10 µg/ml bovine fibronectin (EMD-341631) as described^48^. After three 10-min PBS washes, hydrogels were incubated with cell growth medium for 30 min. Cells were sparsely plated on the hydrogels (~ 1000 cells/cm^2^) and incubated for 24 h before imaging. Fluorescence images of cells and embedded beads were captured at 20X magnification using a Zeiss Axio Observer 7 inverted microscope with Zeiss Axiocam 503 color CCD camera in an environmental chamber at 37 °C and 5% CO_2_. The cells were visualized with Lysosensor Green DND-189 (Invitrogen L7535, 1:1000), added immediately before imaging. Image sequences were taken for live cells and beads. A solution of 10% SDS (Invitrogen; 5% of media volume) was then added to the culture medium and incubated for 10 min before image sequences were taken a second time. Data analysis was performed largely as described^47^ using freely available plug-ins for ImageJ (Tseng et al.^49^; adapted from Dembo and Wang^50^). For Fourier transform traction cytometry, the Poisson’s ratio of the polyacrylamide gel was assumed to be 0.45 and a regularization parameter of 10^−9^ was used. Traction force vector maps were analyzed using a custom script in MATLAB (Mathworks) to determine mean traction stress generated by each cell and total force exerted per cell. Cell boundaries were determined from the corresponding images of green fluorescence in ImageJ. For cells genetically modified with siRNA or adenoviral infection, cells were transfected or infected as described above, incubated in fresh media for 48 h, and plated on hydrogels for an additional 24 h before being imaged for TFM. For blebbistatin treated cells, SMCs were incubated with 15 µM blebbistatin or vehicle (an equivalent dilution of DMSO) in growth media for 30 min prior to traction force microscopy analysis. Results shown for the TFM experiments were accrued from 2 to 3 independent experiments. As variability in traction forces between experiments may arise from batch-to-batch differences in hydrogels or primary SMCs, relevant controls were included as reference in each experiment. Similarly, comparisons between experiments were based on percent changes in mean traction force per cell rather than changes in Newtons.

### Statistical analysis

Statistical analysis was conducted using Prism software (GraphPad) with tests based on the method of data collection and quantification (see Supplemental Methods). Statistical significance for immunostaining, TFM, and RT-qPCR was determined using 2-tailed Mann–Whitney tests unless testing for significance in a particular direction. Significance in immunoblot results was determined by Wilcoxon signed rank test. Significance of the myography vasoconstriction experiments was determined by two-way ANOVA, and statistical differences due to genotype are shown in the figure.

## Supplementary Information

Supplementary Information.

## Data Availability

The datasets analyzed during the current study are available in the GEO Datasets repository under the ascension number GSE165409, [https://www.ncbi.nlm.nih.gov/geo/query/acc.cgi?acc=GSE165409].
