# Supplementary material for: Decreased vascular smooth muscle contractility in Hutchinson–Gilford Progeria Syndrome linked to defective smooth muscle myosin heavy chain expression
Source: Sci Rep. 2021 May 19;11:10625. doi: 10.1038/s41598-021-90119-4 (PMC8134495; doi:10.1038/s41598-021-90119-4)
Supplement: Supplementary file 1 — Supplementary Information. [file 41598_2021_90119_MOESM1_ESM.pdf]

Supplemental methods

RT-qPCR. Descending aortas or carotid arteries were isolated and cleaned from 2-month WT and HGPS mice. Cleaned tissues were stored in RNAlater (Qiagen) at -80ºC for subsequent analysis. Total RNA was isolated with the RNeasy Fibrous Tissue Mini Kit (Qiagen) according to manufacturer's instructions. For carotid analyses, left and right carotids were combined for each mouse. For RT-qPCR of cultured SMCs, RNA was extracted using TriZol reagent (Thermofisher 15596026) according to manufacturer's instructions. Reverse transcription reactions contained 200-500 ng of total RNA. Ten to fifteen percent of the cDNA was subjected to RT-qPCR. Samples were run in technical duplicates on an ABI Prism 7000 sequence detection system or ABI Quantstudio 3 with the following primer-probe sets from ThermoFisher:. Tagln: Mm00441661_g1, Myh11: Mm00443013_m1, Acta2: Mm00725412_s1, Cnn1: Mm00487032_m1. For the RT-qPCR of Myh11 isoforms, custom primer-probe (FAM-labeled) sets were from ThermoFisher: SM-A isoform: Forward CCACAAGGGCAAGAAAGACA, Reverse TTCTTGACGGTTTTCGCATT, Probe CAGCATCACGGGGGAGCTG. SM-B isoform: Forward GTGGCATCCTCCCACAAG, Reverse GGATTGGGTTTGCCTGTAGA, Probe CGCAAGGTCCATCTTTTGCCTAC. SM1 isoform: Forward CTTCGCCCAAGTGACTTTTT, Reverse AAGTGACCATGGGTGCAAAT, Probe AACACTGCTCCCAGAGCGAGC. SM2 isoform: Forward ACGCCCTCAAGAGCAAACT, Reverse GCCCTTCTGGAAGGAACAA, Probe CCCCACAGGAAACTTCGCAGT. Samples from each mouse were accrued, and the final RT-qPCR reactions were performed at one time. Results from each experiment were analyzed using the ddCt method and normalized to the mean of the respective control.

Immunoblotting. For tissue immunoblotting, isolated aortas were thawed, chopped into small pieces and sonicated in lysis buffer (50 mM Tris-HCl pH 8, 250 mM NaCl, 2 mM EDTA, 1% NP-40) containing protease inhibitors (Cell Signaling Technologies 5872S). Lysates were centrifuged for 5 min (4ºC, 15,000 x *g*). The supernatants were collected and quantified with the Bio-Rad Protein

Assay (Catalog #5000006) with a BSA standard. Similar amounts (~25μg) of each sample were diluted into SDS sample buffer (final concentration of 50 mM Tris pH 6.8, 2% SDS, 10% glycerol, 0.01% Bromophenol blue, and 1% β-mercaptoethanol), fractionated on 7.5% SDS polyacrylamide gels, and electrophoretically transferred to nitrocellulose. For immunoblotting of cell lysates, isolated WT and HGPS SMCs were plated at confluence in 60-mm culture dishes for 24 h. Cytoplasmic fractions were collected using NE-PER nuclear cytoplasmic extraction kit (Thermo 79933). For cell experiments using siRNA transfection or adenoviral infection, cells were lysed directly in SDS sample buffer after a 72-h incubation with the siRNA or adenovirus.

Incubations with primary antibodies were performed overnight at 4°C with rocking in TBST (Tris-buffered saline with 0.1% Tween 20) with 5% milk or 2% BSA. Incubation with secondary antibodies were performed for 2 h at room temperature in the same TBST buffer as the primary antibody. The antibodies used were non-muscle myosin 2A: Abcam Ab55456 (1:2000), non-muscle myosin 2B: Cell Signaling 3404 (1:2000), smooth muscle actin: Sigma F3777 (1:1000), smooth muscle myosin heavy chain: Protein Tech 21404-1-AP (1:300), glyceraldehyde-3-phosphate dehydrogenase (GAPDH): Invitrogen MA5-15738 (1:500), focal adhesion kinase: BD Transduction labs 610088 (1:300), and α-tubulin: Santa Cruz SC-8035 (1:300). Bound antibodies were visualized by enhanced chemiluminescence. Blots were stripped and re-probed using Restore Western Blot Stripping Buffer (Thermo 21059).

Immunoblots were quantified in ImageJ relative to one of three loading controls (FAK, α-tubulin, or GAPDH) depending upon the molecular masses of additional probed proteins to minimize stripping and reprobing of blots; see Fig. S2 for validation of loading controls. Immunoblot quantification normalized HGPS or Myh11 siRNA signal intensities to the mean signal intensity of the respective WT control, which was set to 1.0 for each blot in order to combine data from multiple experiments. A Grubbs' test was used to justify exclusion of an outlier.

Tissue Immunostaining. For mouse tissue immunostaining, 2- or 24-month WT and 2-month HGPS male mouse right carotid arteries or descending aortas were perfused with PBS, excised, cleaned to remove excess fat, and fixed in Prefer (Anatech #414). Carotids and aortas were embedded in paraffin, and 5-μm cross sections were prepared. Paraffin sections from mouse and human arteries were deparaffinized and hydrated before antigen unmasking (Vector Labs, H3300) for 25 min at ~100°C). Carotid or aortic cross sections were washed in PBS three times before blocking with 2% BSA in PBS for 15 min, incubated overnight at 4°C with antibodies directed to smooth muscle myosin heavy chain (Protein Tech 21404-1-AP, diluted 1:200 in PBS) or smooth muscle actin (FITC-conjugated; Sigma F3777, diluted 1:300 in PBS). Replicate sections were incubated in parallel with isotype-matched control antibodies. All samples were washed three time with PBS before incubation with a 1:100 dilution of Alexa 594-conjugated isotype-matched secondary antibody (Invitrogen goat anti-rabbit A11012) for two h at room temperature. Sections were then washed three times in PBS followed by addition of Dapi (1:500 dilution in PBS). Slides were briefly washed in PBS and then water before mounting with SlowFade Gold (Thermo, S36936). Results were visualized with a Nikon Eclipse 80i microscope with a QI-Click Qimaging camera. Carotid arteries were imaged at 20x magnification and pseudo-colored green (SM-MHC) or red (SMA). Samples from each mouse were accrued, and the final immunostainings were performed at one time.

Images were quantified using ImageJ. The media of each section was traced using the polygon drawing tool, and its raw integrated density was divided by the area of the outlined media to obtain relative fluorescence intensity. Background intensity, as determined from the isotype-matched control antibodies, was negligible. Relative fluorescence intensity values were plotted relative to the median fluorescence intensity value of the corresponding control for each experiment. A Grubbs' test was used to justify exclusion of an outlier.

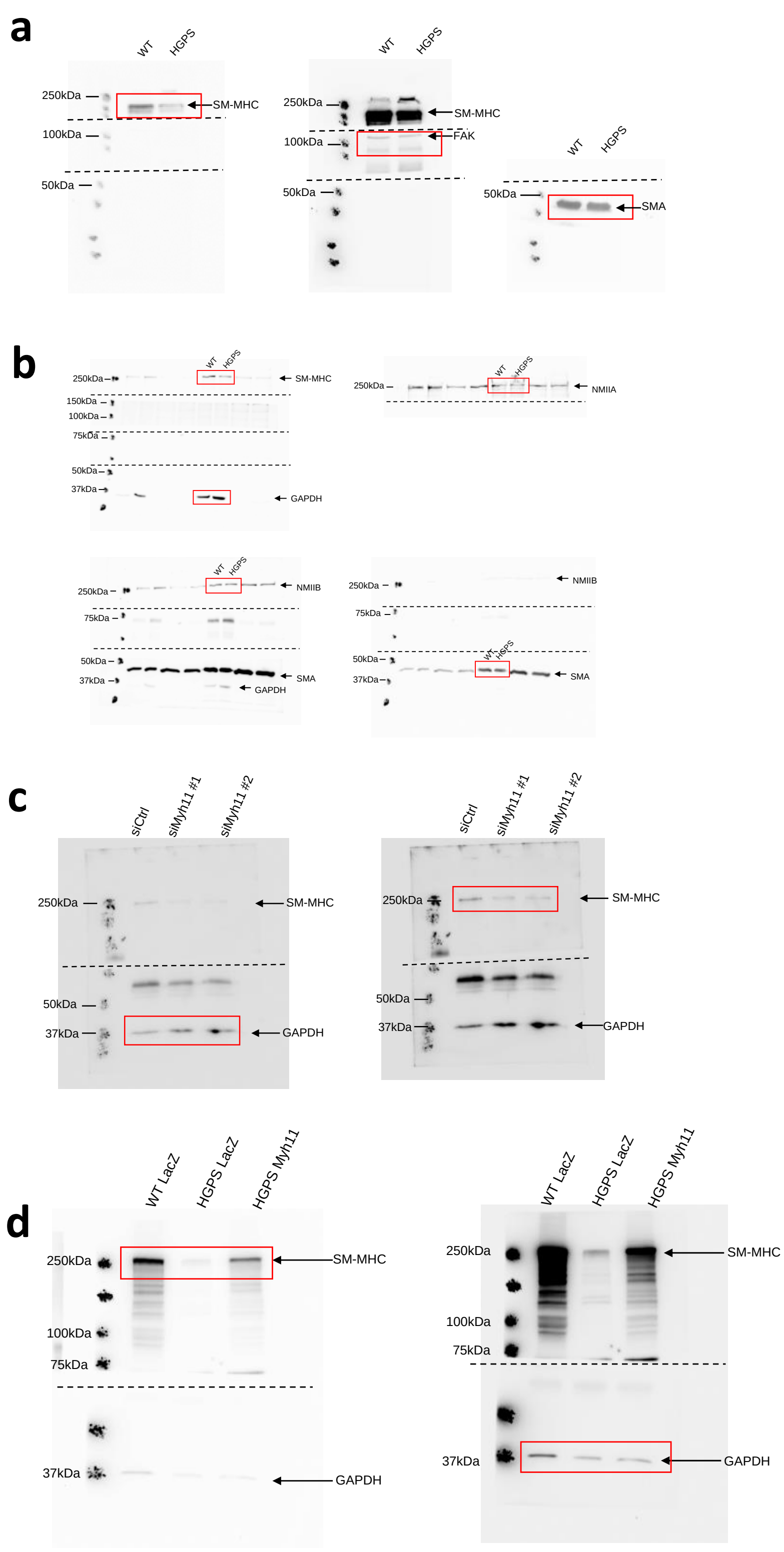

Figure S1. Unprocessed images for immunoblot figures. Nitrocellulose blots were cut as denoted by dashed lines to allow for probing with antibodies to proteins of different molecular mass. Red boxed regions show the cropped area presented in the relevant figure. **(a)** Unprocessed blots for Figure 1d shown at the exposures used in the figure. **(b)** Unprocessed blots for Figure 3a. Filters were probed for SM-MHC and GAPDH, and the exposures used in the figure are shown in red boxes (top left). The 250-kDa portion of the blot used to probe for SM-MHC was also stripped and re-probed; first for NMIIA (top right) and then for NMIIB (bottom left). The blot was also probed for SMA (bottom right) **(c)** Unprocessed blots for Figure 4a shown at the exposures used in the figure. **(d)** Unprocessed blots for Figure 5a shown at the exposures used in the figure. The specific proteins detected are noted with a black arrow on each blot.

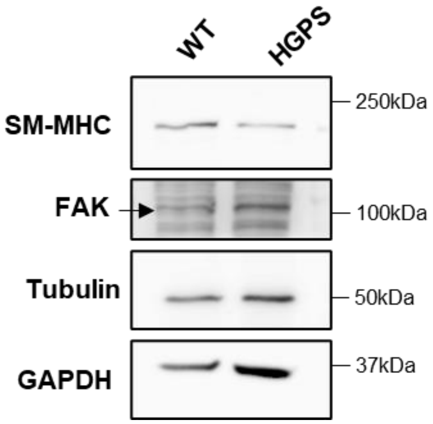

Figure S2. Validation of loading controls. WT and HGPS SMC lysates were immunoblotted for SM-MHC and three loading controls; Focal Adhesion Kinase (FAK), α–Tubulin, and GAPDH. Results are representative of several independent experiments probing for multiple loading controls.
